# Distinct Contributions of Orai1 and TRPC1 to Agonist-Induced [Ca^2+^]_i_ Signals Determine Specificity of Ca^2+^-Dependent Gene Expression

**DOI:** 10.1371/journal.pone.0047146

**Published:** 2012-10-24

**Authors:** Hwei Ling Ong, Shyh-Ing Jang, Indu Suresh Ambudkar

**Affiliations:** Secretory Physiology Section, Molecular Physiology and Therapeutics Branch, National Institute of Dental and Craniofacial Research, National Institutes of Health, Bethesda, Maryland, United States of America; Cornell University, United States of America

## Abstract

Regulation of critical cellular functions, including Ca^2+^-dependent gene expression, is determined by the temporal and spatial aspects of agonist-induced Ca^2+^ signals. Stimulation of cells with physiological concentrations of agonists trigger increases [Ca^2+^]_i_ due to intracellular Ca^2+^ release and Ca^2+^ influx. While Orai1-STIM1 channels account for agonist-stimulated [Ca^2+^]_i_ increase as well as activation of NFAT in cells such as lymphocytes, RBL and mast cells, both Orai1-STIM1 and TRPC1-STIM1 channels contribute to [Ca^2+^]_i_ increases in human submandibular gland (HSG) cells. However, only Orai1-mediated Ca^2+^ entry regulates the activation of NFAT in HSG cells. Since both TRPC1 and Orai1 are activated following internal Ca^2+^ store depletion in these cells, it is not clear how the cells decode individual Ca^2+^ signals generated by the two channels for the regulation of specific cellular functions. Here we have examined the contributions of Orai1 and TRPC1 to carbachol (CCh)-induced [Ca^2+^]_i_ signals and activation of NFAT in single cells. We report that Orai1-mediated Ca^2+^ entry generates [Ca^2+^]_i_ oscillations at different [CCh], ranging from very low to high. In contrast, TRPC1-mediated Ca^2+^ entry generates sustained [Ca^2+^]_i_ elevation at high [CCh] and contributes to frequency of [Ca^2+^]_i_ oscillations at lower [agonist]. More importantly, the two channels are coupled to activation of distinct Ca^2+^ dependent gene expression pathways, consistent with the different patterns of [Ca^2+^]_i_ signals mediated by them. Nuclear translocation of NFAT and NFAT-dependent gene expression display “all-or-none” activation that is exclusively driven by local [Ca^2+^]_i_ generated by Orai1, independent of global [Ca^2+^]_i_ changes or TRPC1-mediated Ca^2+^ entry. In contrast, Ca^2+^ entry via TRPC1 primarily regulates NFκB-mediated gene expression. Together, these findings reveal that Orai1 and TRPC1 mediate distinct local and global Ca^2+^ signals following agonist stimulation of cells, which determine the functional specificity of the channels in activating different Ca^2+^-dependent gene expression pathways.

## Introduction

Stimulation of cells with physiologically relevant agonists that target G protein- or tyrosine kinase-coupled receptors leads to increases in cytosolic [Ca^2+^] ([Ca^2+^]_i_) as a result of inositol 1,4,5-triphosphate (IP_3_)-induced Ca^2+^ release from intracellular Ca^2+^ stores via the IP_3_ receptors (IP_3_Rs) and Ca^2+^ influx via plasma membrane Ca^2+^ channels. The temporal and spatial pattern of [Ca^2+^]_i_ signals generated in response to agonist stimulation are utilized by the cells to regulate various critical functions, such as gene expression, ion channel activation and fluid secretion [1], [2]. High levels of agonist typically induce sustained elevations in baseline [Ca^2+^]_i_, whereas lower [agonist] elicit oscillatory [Ca^2+^]_i_ responses [1], [3], [4] . Two types of oscillations are seen; baseline oscillations that are usually seen at very low [agonist] or oscillations over a sustained elevation in baseline [Ca^2+^]_i_ at relatively higher [agonist]. Such oscillatory responses have been proposed to represent the physiological mode of signaling in many cell types and have been observed in almost all cell types, including cell lines as well as primary cell preparations from various tissues [3], [5], [6], [7], [8]. These oscillations primarily reflect repetitive cycles of Ca^2+^ release from the ER stores via IP_3_Rs, inhibition of Ca^2+^ release, and reuptake into the store due to SERCA pump activity. In several cell types, sustained [Ca^2+^]_i_ oscillations require extracellular Ca^2+^ influx to achieve refilling of the ER store after every release event, thus priming it for the next release cycle. Even in cells where the oscillations are sustained for longer periods in the absence of external Ca^2+^, intracellular Ca^2+^ stores are eventually depleted and there is a run-down of [Ca^2+^]_i_ oscillations.

Store-operated calcium entry (SOCE) is activated in response to depletion of Ca^2+^ within the ER as a result of IP_3_-induced Ca^2+^ release following agonist stimulation of cells [1], [9]. SOCE has been shown to the primary determinant of agonist-induced [Ca^2+^]_i_ oscillations in a number of cells. Removal of extracellular Ca^2+^, or inhibition of Ca^2+^ influx with La^3+^, induced cessation of [Ca^2+^]_i_ oscillations [5], [10], [11]. A major component of SOCE is STIM1, an ER Ca^2+^ binding protein that serves as a sensor for ER-[Ca^2+^] and regulates plasma membrane calcium channels mediating SOCE. [12], [13], [14]. Orai1, the pore-forming subunit of the highly Ca^2+^-selective Ca^2+^ release-activated Ca^2+^ (CRAC) channel has now been established as an essential component of SOCE [15], [16], [17]. Orai1 determines critical cellular functions including T-lymphocyte activation and mast cell degranulation. Suppression of Orai1 or STIM1 expression or function leads to elimination of SOCE and CRAC channel function [12], [13], [15], [18], [19]. Transient receptor potential 1 (TRPC1) is also activated in response to stimulation of cells by agonists or thapsigargin (Tg), and is a major contributor to Ca^2+^ influx in some cell types [1], [2], [20], [21], [22], [23], [24]. We have previously shown that TRPC1 forms a dynamic complex with STIM1 and Orai1 in response to store depletion [25], [26], [27]. Further, data from several laboratories have demonstrated that while TRPC1 is gated by STIM1, its function depends on Orai1 [26], [27], [28], [29]. Our recent finding provide evidence that Orai1-mediated Ca^2+^ entry triggers recruitment of TRPC1 into the plasma membrane where it is activated by STIM1 [27]. However, once activated, the two channels appear to have distinct functional contributions. In TRPC1−/− mice, decrease in Ca^2+^ entry in salivary acinar cells is associated with loss of salivary fluid secretion as well as Ca^2+^-dependent K^+^ channel activation [20]. These findings suggest that TRPC1 generates [Ca^2+^]_i_ signals that are specifically required for the activation of K_Ca_ channels in acinar cells, which cannot be achieved or are not compensated for by the residual Orai1 channel in these cells. A similar decrease in Ca^2+^-activated Cl^-^ channel activity has been shown in pancreatic acini from TRPC1−/− mice [8].

CRAC channel activity has been associated with [Ca^2+^]_i_ oscillations in RBL cells and T lymphocytes [30], [31], [32], [33]. Importantly, CRAC channel-mediated [Ca^2+^]_i_ oscillations have been shown to underlie regulation of Ca^2+^-dependent gene expression via activation of the transcription factor, nuclear factor activated T cells (NFAT). NFAT is translocated in an “all-or-none” manner following its activation which involves its complete dephosphorylation by calcineurin, a Ca^2+^-CaM dependent phosphatase [5]. By contrast, the nuclear factor kappa-light-chain enhancer of activated B cells (NFκB) pathway is activated through DAG- and Ca^2+^-dependent degradation of the inhibitor of NFκB (IκB), and is suggested to exhibit a strong dependence on peak amplitude, rather than duration, of a [Ca^2+^]_i_ signal. In B cells, a high level of [Ca^2+^]_i_, exceeding that achieved by CRAC channel activity alone, is required for activation of NFκB. [34].

Our previous study suggested that Orai1-mediated Ca^2+^ influx is more relevant for NFAT-activation than TRPC1, but is less relevant for K_Ca_ activation. However, both TRPC1 and Orai1 are required for NFκB activation [27]. Since both Orai1-STIM1 and TRPC1-STIM1 channels contribute to agonist- or Tg-stimulated [Ca^2+^]_i_ increases in human submandibular gland (HSG) cells, but only Orai1-mediated Ca^2+^ entry regulates the activation of NFAT, it is unclear how the cells decode individual Ca^2+^ signals generated by the two channels for the regulation of specific cellular functions. We hypothesized that the two channels induce distinct [Ca^2+^]_i_ signals which determine their functional specificity in the regulation of Ca^2+^-dependent gene expression. To assess this, we have examined the contributions of Orai1 and TRPC1 to carbachol (CCh)-induced [Ca^2+^]_i_ signals and Ca^2+^-dependent gene expression in single cells. Herein we report that endogenous Orai1 and TRPC1 channels contribute to distinct local and global [Ca^2+^]_i_ signals following agonist stimulation of salivary gland epithelial cells. Increasing concentrations of a physiologically relevant agonist, CCh (0.3 µM to 100 µM) induced baseline [Ca^2+^]_i_ oscillations, oscillations over a sustained baseline elevation, or sustained baseline elevation without oscillations. While suppression of Orai1 expression or function completely eliminated Ca^2+^ influx at all [CCh], inhibition of TRPC1 reduced sustained [Ca^2+^]_i_ elevation and decreased the frequency of residual [Ca^2+^]_i_ oscillations. Thus, Orai1 channel function is the primary determinant of [Ca^2+^]_i_ oscillations. Importantly, at any [agonist] and irrespective of TRPC1 contribution to [Ca^2+^]_i_, “all-or-none” activation of NFAT was solely dependent on Orai1 and did not reflect global [Ca^2+^]_i_. In contrast, TRPC1 had a substantial contribution to activation of NFκB-dependent gene expression. In aggregate, these novel findings demonstrate that Orai1 and TRPC1 mediate distinct and specific [Ca^2+^]_i_ signals following agonist stimulation that determine their differential activation of Ca^2+^-dependent transcription factors in a single cell.

## Results

### Orai1 and TRPC1 Channels Contribute Distinct [Ca^2+^]_i_ Signals in a Single Cell

**Figure 1:**
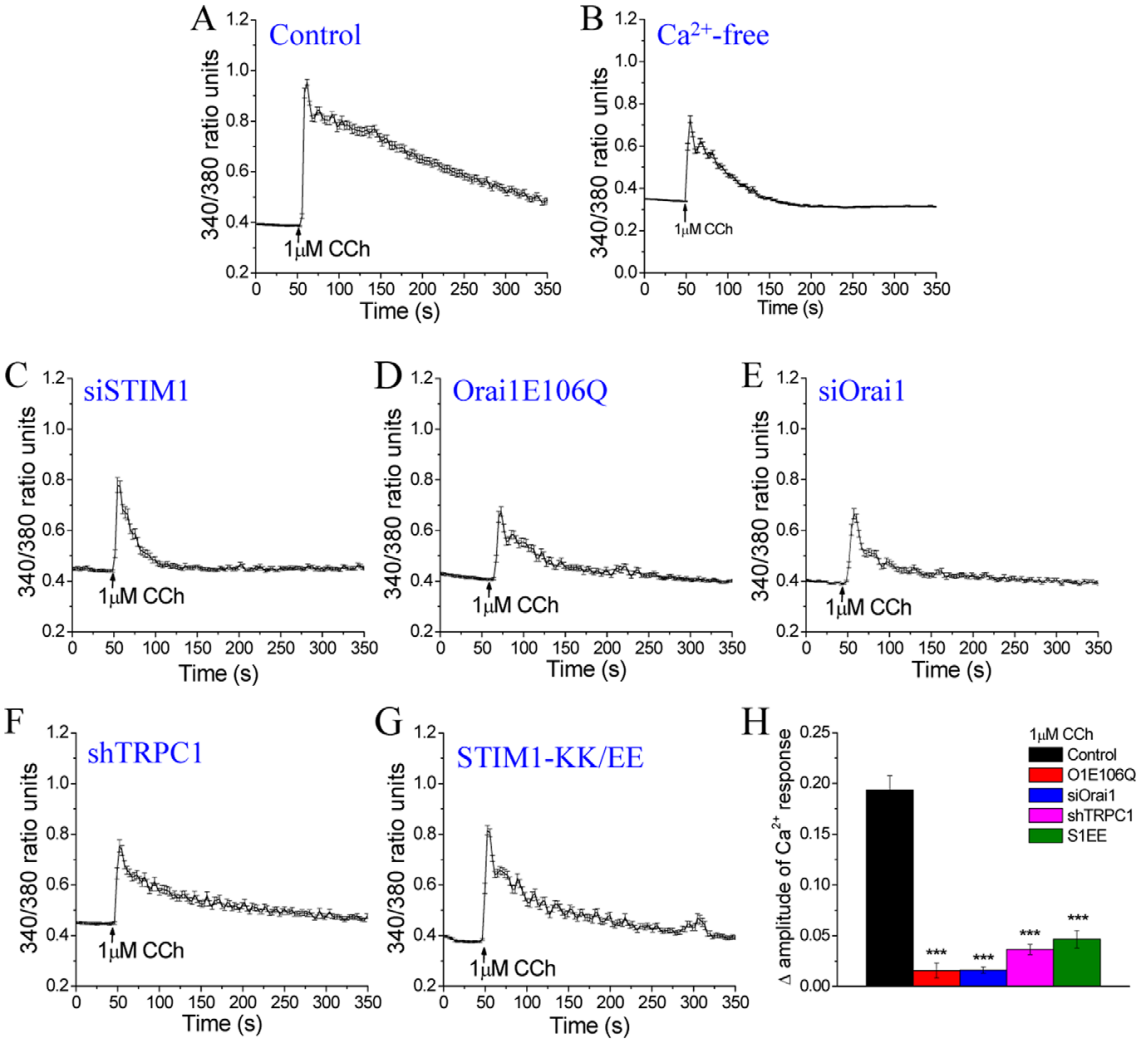
SOCE-driven [Ca^2+^]_i_ increases in HSG cells stimulated with relatively low [CCh]. Averaged [Ca^2+^]_i_ responses induced by relatively low [CCh] (1 µM) in control HSG cells, with and without extracellular Ca^2+^ (**A, B**), and cells expressing siSTIM1 (**C**), Orai1E106Q (**D**), siOrai1 (**E**), shTRPC1 (**F**), or STIM1-KK/EE (**G**). Data for each trace were obtained from ≥50 cells in at least 3 separate experiments. (**H)** Average data showing amplitude of [Ca^2+^]_i_ increase at t = 250 s (F_t_−F_0_). *** indicates a significant difference (P<0.001, n ≥ 80 cells).

Following CCh stimulation of HSG cells, a fairly sustained [Ca^2+^]_i_ elevation was seen, with an initial rapid increase followed by a sustained elevation above baseline that slowly declined over time (Figure 1A shows the average response in a cell population). In the absence of external Ca^2+^, only a transient increase in [Ca^2+^]_i_ was seen (Figure 1B), suggesting that the sustained [Ca^2+^]_i_ increase following agonist stimulation is dependent on extracellular Ca^2+^ entry. Knockdown of STIM1 expression by siSTIM1 eliminated the sustained [Ca^2+^]_i_ elevation, producing a pattern similar to that in the absence of external Ca^2+^ (Figure 1C). Complete elimination of sustained [Ca^2+^]_i_ elevation was also seen with expression of Orai1E106Q (a dominant negative Orai1 mutant, [35], [36]) or siOrai1 (>90% decrease; Figure 1, D, E and H). On the other hand, expression of shTRPC1 or STIM1-KK/EE [29] induced >60% decrease in sustained [Ca^2+^]_i_ elevation (responses at 250 s were 0.046±0.009 and 0.062±0.008, respectively, which are both significantly higher than that in Orai1E106Q- or siOrai1-expressing cells (0.016±0.007 and 0.016±0.003 respectively), and lower than in control cells (0.193±0.014) (*p<*0.001; Figure 1, F to H). Based on our previous studies, we can conclude that cells expressing siOrai1 or Orai1E106Q lack both TRPC1 and Orai1 functions [26], [27]. While these findings demonstrate that Ca^2+^ entry via Orai1 and TRPC1 determine sustained the [Ca^2+^]_i_ elevation seen in CCh-stimulated cells, the individual contributions of the two channels cannot be resolved using such measurements.

**Figure 2:**
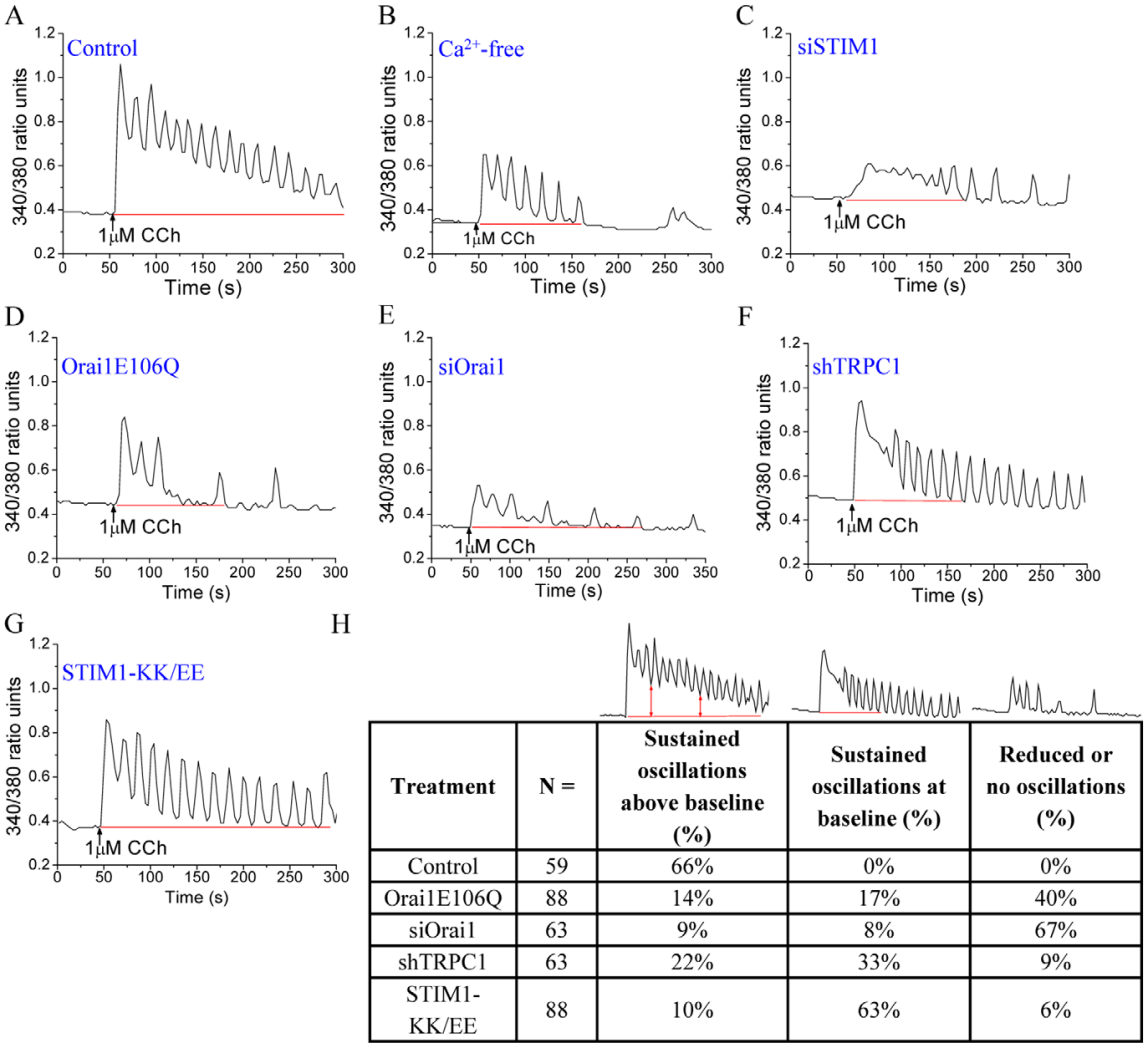
TRPC1 and Orai1 contribute distinct [Ca^2+^]_i_ changes in individual HSG cells following CCh stimulation. Ca^2+^ oscillations in single cells following relatively low [CCh] (1 µM) stimulation of control HSG cells, with and without extracellular Ca^2+^ (**A** and **B**); or cells expressing siSTIM1 (**C**), Orai1E106Q (**D**), siOrai1 (**E),** shTRPC1 (**F**) or STIM1-KK/EE (**G**). Each trace is representative of ≥50 cells in at least 3 separate experiments (all traces shown were obtained from a single experiment). (**H**) Different oscillatory patterns seen in control cells as well as the changes induced by loss of TRPC1 or Orai1 function. Cells showing the various oscillatory patterns were counted and shown as percentage (%) of total cells (N). Cells showing sustained, non-oscillatory responses, or no responses were excluded from the data.

To determine the characteristics of [Ca^2+^]_i_ signals generated by Orai1 and TRPC1, we carried out detailed analysis of the [Ca^2+^]_i_ changes induced by CCh in individual HSG cells. Stimulation with 1 µM CCh induced a rapid initial increase [Ca^2+^]_i_ which was followed by oscillatory increases over a sustained elevation of baseline [Ca^2+^]_i_ (Figure 2A). The initial response primarily represents Ca^2+^ release from the ER, while the subsequent oscillatory responses and sustained baseline elevation are determined by the influx of Ca^2+^ (*c.f.* trace in absence of external Ca^2+^, Figure 2B). Knockdown of STIM1 using siRNA (siSTIM1) reduced both the oscillations and sustained elevation of baseline [Ca^2+^]_i_ (Figure 2C), providing evidence for the involvement of SOCE in sustaining the [Ca^2+^]_i_ oscillations in these cells. To resolve the individual [Ca^2+^]_i_ signals contributed by TRPC1 and Orai1, the pattern of [Ca^2+^]_i_ signals was examined in cells expressing either shTRPC1 or STIM1-KK/EE (residual activity is determined by Orai1) or in cells expressing siOrai1 or Orai1E106Q (both TRPC1 and Orai1 functions are attenuated). Suppression of both Orai1 and TRPC1 functions induced an overall reduction in [Ca^2+^]_i_ elevation, similar to that seen with siSTIM1, with abrogation of both the oscillatory responses as well as sustained [Ca^2+^]_i_ elevation (Figure 2, D and E). Importantly, suppression of TRPC1 function resulted in loss of the baseline elevation in [Ca^2+^]_i_ and converted the pattern to baseline oscillations that are driven by Orai1-mediated Ca^2+^ entry (Figure 2, F and G).

The relative proportions of cells affected by these maneuvers are shown in Figure 2H. In the control group, 66% of cells showed oscillatory [Ca^2+^]_i_ responses over a sustained elevation in baseline [Ca^2+^]_i_ while none of the cells displayed sustained baseline oscillations or transient responses (cells that did not respond or those that showed a sustained elevation without oscillations were not included). Interestingly in cells expressing STIM1-KK/EE, the proportion of cells with [Ca^2+^]_i_ oscillations over an elevated baseline decreased to 10% (from 66% in control cells) and those with baseline oscillations increased to 63%, whereas only 6% of cells showed transient responses. Similar changes were seen in cells expressing shTRPC1. In the case of Orai1E106Q- and siOrai1-expressing cells, the proportion of cells with transient response was increased to 40% or 67%, respectively, as would be expected if the SOCE process was inhibited (about 14% or 9% of cells displayed normal response and 15% or 8% showed baseline oscillations, these likely represent either non-transfected cells or those expressing low levels of Orai1 mutant or siOrai1 respectively).

In aggregate, the data in Figure 2 demonstrate that Ca^2+^ entry via TRPC1 and Orai1 channels generate distinct cytosolic Ca^2+^ signals. Orai1 primarily controls oscillatory [Ca^2+^]_i_ signals while TRPC1-mediated Ca^2+^ entry generates a more sustained elevation of [Ca^2+^]_i_. These contributions of Orai1 and TRPC1 to CCh-stimulated [Ca^2+^]_i_ signals were confirmed in HEK293 cells. HEK293 cells displayed baseline oscillatory [Ca^2+^]_i_ responses following stimulation with 1 µM CCh, which were primarily dependent on the STIM1-activated Orai1 channel (Figure S1A). When TRPC1 and STIM1 were co-expressed in HEK293 cells, the pattern of baseline [Ca^2+^]_i_ oscillations was converted to oscillations over a sustained elevation (Figure S1B). In contrast, expression of STIM1-KK/EE in HEK293 cells did not significantly alter CCh-induced [Ca^2+^]_i_ oscillations (Figure S1C; *c.f.* control cells in Figure S1A). Furthermore, as described earlier for HSG cells, these CCh-induced oscillations in HEK293 cells were dependent on Ca^2+^ entry and could not be sustained with the absence of Ca^2+^ in the extracellular milieu (Figure S1D). Thus, when both Orai1 and TRPC1 are activated simultaneously, the typical response achieved is oscillations over a sustained elevation of baseline [Ca^2+^]_i_. While both channels contribute to SOCE in HSG cells, the Orai1 channel predominantly mediates SOCE in HEK293 cells. Together, the data discussed above suggest that inclusion of functional TRPC1 channels leads to modulation of the [Ca^2+^]_i_ signals generated by Orai1 channels.

### NFAT is Regulated by Orai1-mediated Ca^2+^ Influx

**Figure 3:**
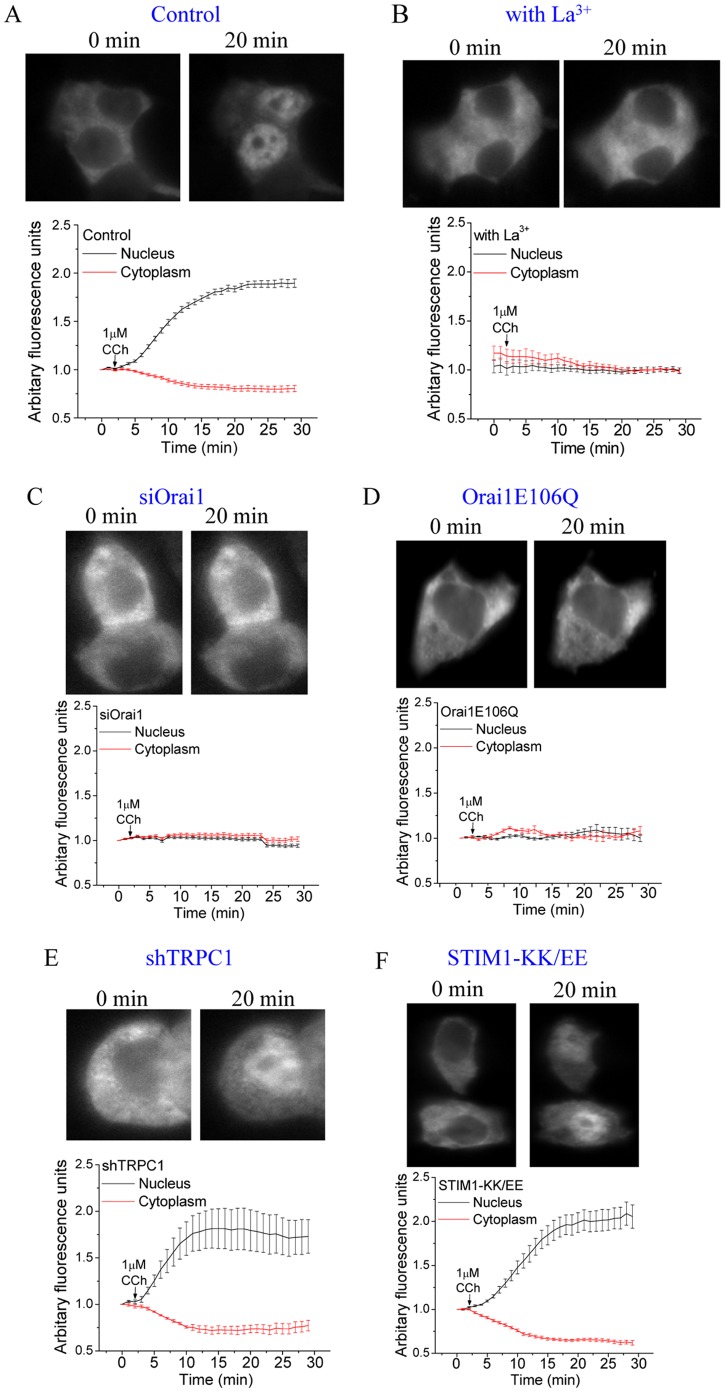
Control of translocation of NFAT into the nucleus in CCh-stimulated cells. Translocation of NFAT into the nucleus in control cells (**A**), cells in medium containing 1 mM La^3+^ (**B**), and cells transfected with siOrai1 (**C**), Orai1E106Q (**D**), shTRPC1(**E**) and STIM1EE (**F**). Traces show changes in GFP fluorescence intensities within the nucleus (black) and cytoplasm (red), following stimulation with 1 µM CCh. Each trace is representative of at ≥30 cells in at least 3 separate experiments.

We then investigated whether the cellular mechanisms regulating activation of NFAT can decode the different [Ca^2+^]_i_ signals generated by Ca^2+^ entry via Orai1 and TRPC1 channels. Translocation of GFP-NFAT into the nucleus, with a corresponding decrease of protein in the cytosol, was detected within 2 min of stimulation with low [CCh] (1 µM), reaching a maximum at about 20 min which was maintained during the period of the experiment (60 min) (Figure 3A). NFAT activation was exclusively driven by Ca^2+^ entering the cell since application of 1 mM La^3+^ extracellularly blocked nuclear translocation of GFP-NFAT following stimulation with 1 µM CCh (Figure 3B). At this concentration, La^3+^ blocks both Ca^2+^ entry via plasma membrane Ca^2+^ channels as well as Ca^2+^ extrusion via the plasma membrane Ca^2+^-ATPase pump thereby retaining the Ca^2+^ released from the ER in the cytoplasm. NFAT translocation into the nucleus was also abrogated in cells where Orai1 function was suppressed (compare A to C and D in Figure 3). However, in cells where only TRPC1 expression was suppressed, the residual Orai1 activity was sufficient to support activation of NFAT (Figure 3E and F). We had reported similar specificity for Orai1 in NFAT-dependent gene expression following Tg stimulation of HSG cells [27]. Together the data in Figures 1, 2 and 3 show that while Ca^2+^ entry via both Orai1 and TRPC1 channels contribute to CCh-induced [Ca^2+^]_i_ elevation, only the [Ca^2+^]_i_ signal generated by Orai1 is decoded by the cells for activation of NFAT. We suggest that Ca^2+^ entry, and probably the resulting local [Ca^2+^]_i_ microdomain near the Orai1 channel, is involved in this regulation.

### Specificity of [Ca^2+^]_i_ Signals and NFAT Regulation in Cells Stimulated with Higher Level of Agonist

**Figure 4:**
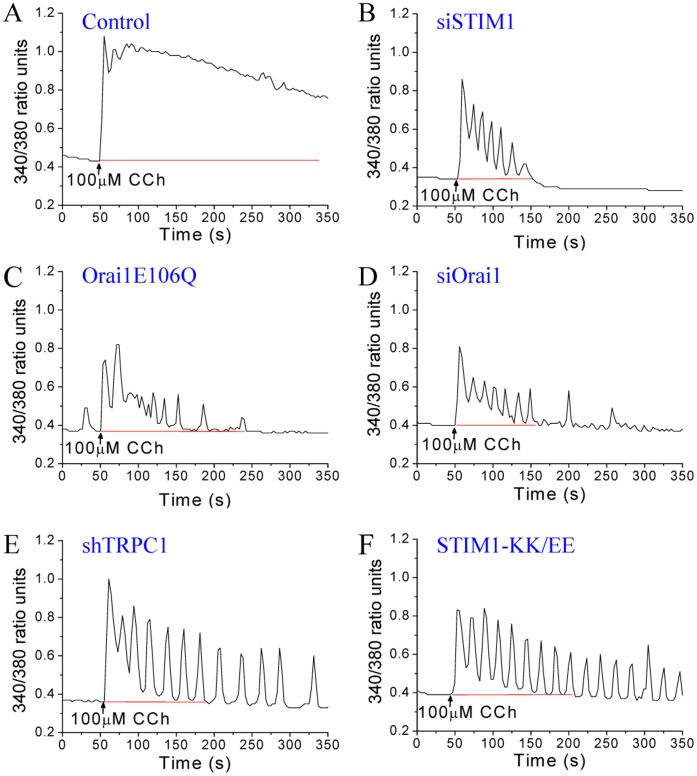
Contribution of Orai1 and TRPC1 to [Ca^2+^]_i_ signals in individual cells stimulated at high [CCh]. [Ca^2+^]_i_ increases induced by high [CCh] (100 µM) in control cells (**A**), and in cells transfected with siSTIM1 (**B**), Orai1E106Q (**C**), siOrai1 (**D**), shTRPC1 (**E**), or STIM1-KK/EE (**F**). Traces are from a single experiment and are representative of ≥50 cells in at least 3 separate experiments.

Stimulation of cells with higher [agonist] can be expected to induce a different spatial pattern of [Ca^2+^]_i_ signals. Thus, we measured [Ca^2+^]_i_ changes elicited by relatively high [CCh] (100 µM) in cell populations, as well as in individual cells. In control cells, there was higher and more sustained [Ca^2+^]_i_ elevation with <10% of the cells showing any type of oscillations (Figure 4A; trace showing average data from >50 cells is in Figure S2A). Following knockdown of STIM1, majority of the cells displayed a transient [Ca^2+^]_i_ increase (Figure 4B) with a significant reduction (>80%) in the magnitude of sustained [Ca^2+^]_i_ elevation (Figure S2B). Similarly, suppression of Orai1 function led to a transient [Ca^2+^]_i_ response where majority of the cells showing reduced or no oscillations (Figure 4, C and D), with a significantly reduced magnitude of sustained [Ca^2+^]_i_ elevation (Figure S2, C, D and G). Cells expressing shTRPC1 or STIM1-KK/EE showed a ∼60% decrease in the sustained [Ca^2+^]_i_ elevation (Figure S2, E to G). However, at a single cell level, there was a marked effect of TRPC1 suppression on the pattern [Ca^2+^]_i_ increase. The sustained [Ca^2+^]_i_ elevation seen in control cells was converted to baseline oscillations (seen in ≥50% of the cells; Figure 4, E and F). Thus, we conclude that the residual Orai1-mediated Ca^2+^ entry drives an oscillatory [Ca^2+^]_i_ signal in cells stimulated with either high and relatively low [agonist].

**Figure 5:**
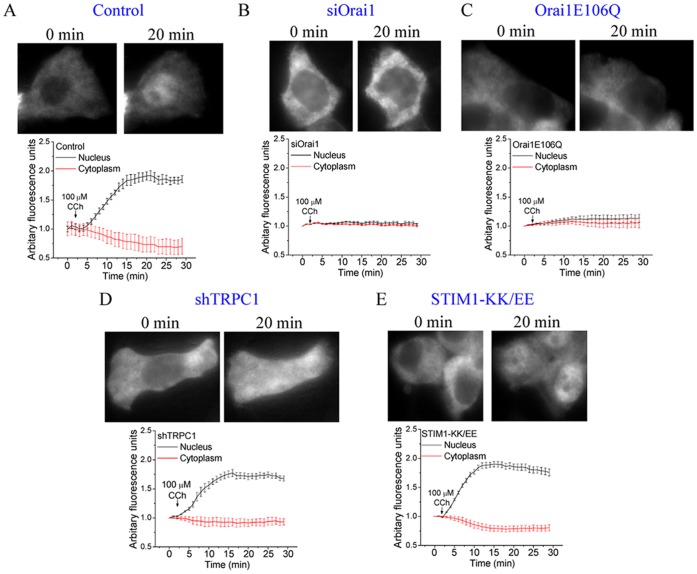
Effect of high [CCh] on NFAT nuclear translocation. Translocation of NFAT into the nucleus in control cells (**A**), and cells transfected with siOrai1 (**B**), Orai1E106Q (**C**), shTRPC1 (**D**) and STIM1EE (**E**). Traces show changes in GFP fluorescence intensities within the nucleus (black) and cytoplasm (red), following stimulation with 100 µM CCh. Each trace is representative of data obtained from at least 3 separate experiments (≥ 30 cells).

NFAT activation was also assessed in single cells stimulated with 100 µM CCh. Nuclear translocation of NFAT was not faster than that in cells stimulated with 1 µM CCh, although the number of cells responding to the stimulus was significantly higher (Figure 5A *c.f.* Figure 3A, >90% cells vs. ∼70% cells displayed NFAT translocation). Further, as seen in cells stimulated with 1 µM CCh, NFAT translocation was completely prevented by expression of Orai1E106Q and siOrai1 but was not affected by STIM1-KK/EE and shTRPC1 (Figure 5, B to E). Thus, even at relatively high levels of stimuli, where global [Ca^2+^]_i_ changes were markedly different from that induced by lower [CCh], NFAT activation was solely driven by Orai1-mediated Ca^2+^ entry. These data also reveal a very important aspect of the regulation of NFAT, i.e. this transcription factor is regulated by local [Ca^2+^]_i_ signals generated by Orai1 and is not affected by global [Ca^2+^]_i_ or by [Ca^2+^]_i_ signals contributed by TRPC1.

### Stimulation with Low [CCh] Reveals Orai1-dependent “All-or-none” Pattern of NFAT Activation

**Figure 6:**
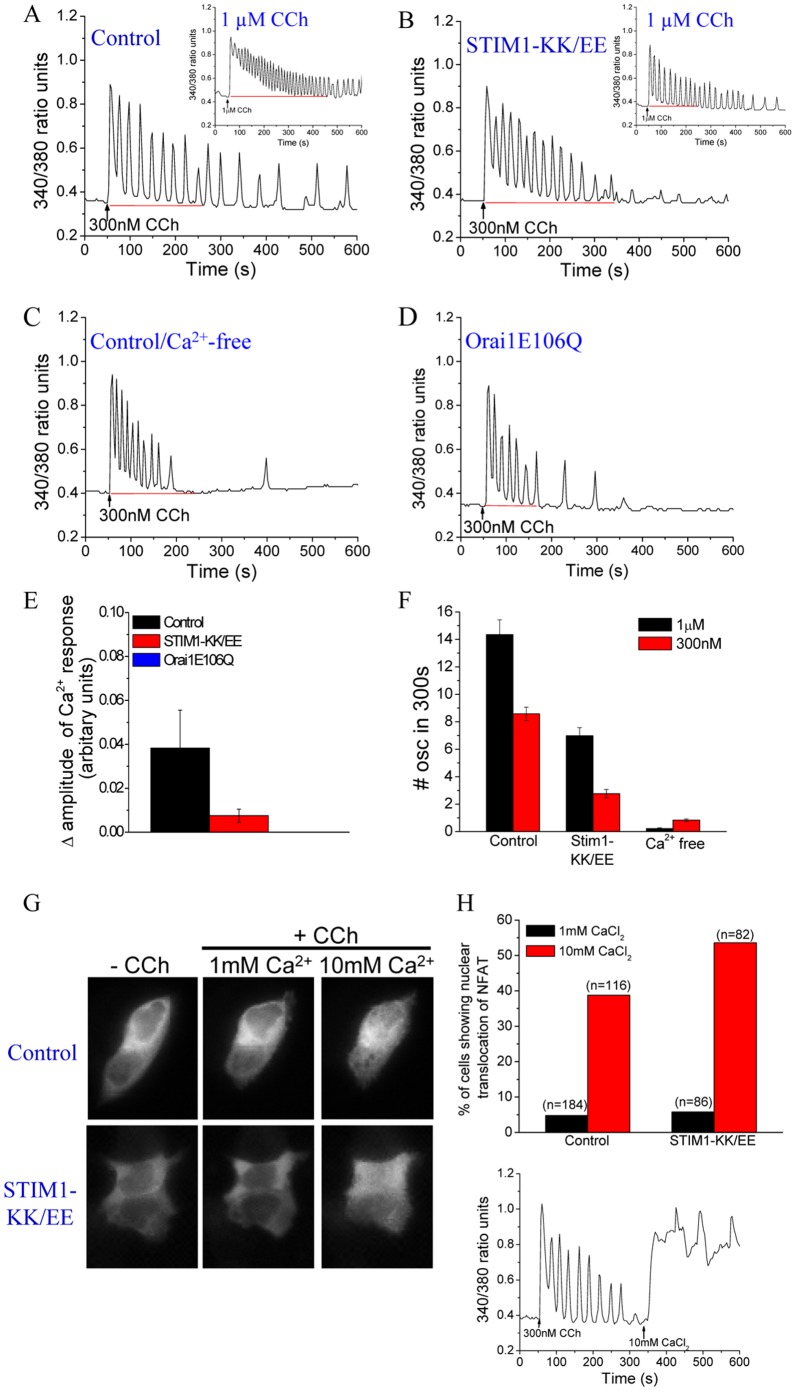
“All-or-none” mode of NFAT activation by Orai1-mediated Ca^2+^ entry. [ Ca^2+^]_i_ responses induced by 300 nM CCh in control cells (**A**), cells expressing STIM1-KK/EE (**B**) or Orai1E106Q (**D**), and cells in Ca^2+^-free media (**C**). Each trace is representative of data obtained from ≥50 cells in at least 3 separate experiments. Insets in A and B show corresponding responses induced by 1 µM CCh over a 10 min time period. (**E**) Average change in the amplitude of [Ca^2+^]_i_ at t = 350 s (F_t_−F_0_). (**F**) Number of oscillations between the 300 and 600 s time points in control cells, cells expressing STIM1-KK/EE or cells in Ca^2+^-free media following stimulation with 1 µM or 300 nM CCh. (**G**) Panels showing nuclear translocation of NFAT in control cells and cells expressing STIM1-KK/EE, following stimulation with 300 nM CCh with 1 and 10 mM extracellular CaCl_2_. (**H**) Histogram showing the proportion of cells (%) showing nuclear translocation of NFAT following stimulation with 300 nM CCh with 1 and 10 mM extracellular CaCl_2_. Trace shows the **[**Ca^2+^]_i_ responses induced by 300 nM CCh in the presence of 1 and 10 mM extracellular CaCl_2_, and is representative of data obtained from ≥50 cells in at least 3 separate experiments.

At very low [CCh] (300 nM), only about 30% of the cells responded by displaying sustained baseline oscillations (Figure 6A). Initial oscillations due to intracellular Ca^2+^ release (cells stimulated in Ca^2+^-free medium) were slightly more prolonged (up to 250 s; Figure 6C; similar results were obtained in cells expressing Orai1E106Q in Figure 6D) probably due to the low levels of Ca^2+^ store depletion. In order to determine the Ca^2+^ influx component, [Ca^2+^]_i_ was monitored for a period of 10 min and the value at 350 s was used to determine the amplitude. The number of oscillations generated between 300–600 s were also counted (note that oscillations subside by this time in a Ca^2+^-free medium). Importantly at this low level of stimulation, there was a detectable contribution of TRPC1 to the frequency of [Ca^2+^]_i_ oscillations as cells expressing STIM1-KK/EE showed a rundown of oscillations with >75% decrease in the number of oscillations (Figure 6, B, E and F). Based on these findings, we examined the [Ca^2+^]_i_ oscillations in cells stimulated at 1 µM CCh between 300–600 s (representative traces are shown in insets of Figure 6A and B). As shown in Figure 6F, there was ∼50% decrease in the frequency of oscillations due to suppression of TRPC1 activity in cells stimulated at 1 µM CCh, *c.f.* ∼62% decrease with 300 nM CCh. Thus, Ca^2+^ influx via TRPC1 also contributes to [Ca^2+^]_i_ oscillations. We suggest that this is likely due to more efficient refilling of the Ca^2+^ stores in cells when both Orai1 and TRPC1 channels are active.

A major finding of this study was the dissociation between [Ca^2+^]_i_ changes and NFAT activation seen in cells stimulated with 300 nM CCh. Despite detection of [Ca^2+^]_i_ oscillations in 30% of the cells, only 4.8% of the cells exhibited nuclear translocation of NFAT (compare with >90% and ∼70% of cells stimulated with 100 or 1 µM CCh, respectively). Since the number of oscillations at this very low [CCh] is about 40–50% less and amplitude of the [Ca^2+^]_i_ signal is ∼75% lower than that at 1 µM CCh (Figure 6E and F, *c.f.* Figure 1H), we hypothesized that a lower number of Orai1 channels are activated and therefore, the local increase in [Ca^2+^]_i_ is lower than the threshold required for NFAT activation. To test this, extracellular [Ca^2+^] was raised from 1 to 10 mM to increase the driving force for Ca^2+^ entry via Orai1, which should increase the local [Ca^2+^]_i_ near the channel. Consistent with our prediction, this maneuver resulted in a significant increase in the number of cells exhibiting nuclear translocation of NFAT to 38.8% (Figure 6G and H). Similarly, NFAT translocation was seen in very few cells expressing STIM1-KK/EE unless extracellular [Ca^2+^] was increased to 10 mM (5.8% at 1 mM to 53.6% at 10 mM Ca^2+^; Figure 6G and H). Furthermore, a sustained elevation of [Ca^2+^]_i_ with minimal oscillations was seen when extracellular [Ca^2+^] was increased to 10 mM (Figure 6H). In aggregate, these findings further establish that only local [Ca^2+^]_i_ near Orai1 channels is involved in NFAT activation.

A recent study showed that there is a threshold for local [Ca^2+^]_i_ generated by Orai1-mediated Ca^2+^ influx which is critical for dephosphorylation of NFAT [37]. These investigators showed that while stimulation with 120 nM leukotriene C_4_ (LTC_4_) was insufficient to induce activation of NFAT1, a second pulse of 120 nM LTC_4_ (total = 240 nM) within 10 min of the first was sufficient to activate NFAT1. We have observed similar results when two pulses of 300 nM CCh within 3 min of each other were added to the cells. The number of cells showing nuclear translocation of NFAT increased from about 4.35% (n = 184) to 11.7% (n = 171). Furthermore, this was not altered by suppression of TRPC1 function in STIM1-KK/EE-expressing cells, where 14.4% of cells (n = 118) showed NFAT activation following two pulses of 300 nM CCh (*c.f.* 5.8% (n = 86) for a single pulse). We suggest that the first pulse of CCh was not sufficient to completely dephosphorylate NFAT, while the second pulse of agonist achieved the extra [Ca^2+^]_i_ increase needed to fully dephosphorylate NFAT, a requirement for its nuclear translocation. Collectively, the data presented in Figure 6 demonstrate that although TRPC1 contributes to oscillatory [Ca^2+^]_i_ responses at very low [agonist], nuclear translocation of NFAT is solely dependent on local [Ca^2+^]_i_ signals generated by Orai1. Further, the lack of NFAT translocation at low levels of stimuli when compared to the relatively higher levels, suggests that local [Ca^2+^]_i_ required to drive this process is likely to depend on the number of Orai1 channels activated at any given [agonist] and that NFAT regulation is mediated by an “all-or-none” mechanism in which complete dephosphorylation of the transcription factor is required for its nuclear translocation. What is more important is that TRPC1-mediated Ca^2+^ entry does not modulate the local [Ca^2+^]_i_ near Orai1, although it increases global [Ca^2+^]_i_ and contributes to the frequency of [Ca^2+^]_i_ oscillations.

### Specific Regulation of NFAT- and NFκB-driven Luciferase Activities by Orai1 and TRPC1 Channels

**Figure 7:**
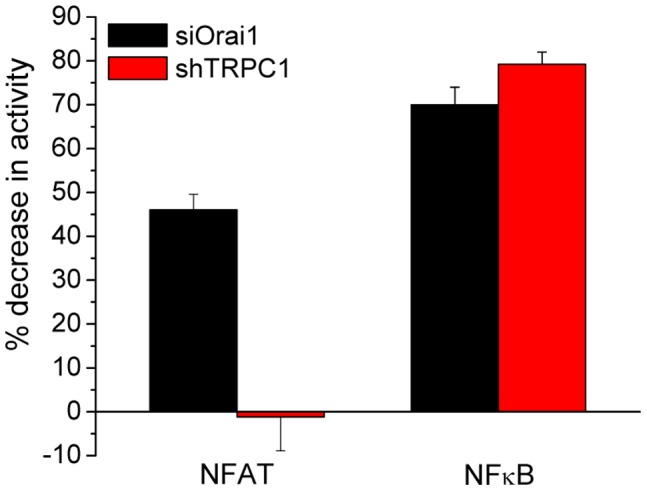
Contribution of Orai1 and TRPC1 channels to the activation NFAT- and NFκB-driven luciferase activities. Luciferase activities driven by NFAT and NFκB following stimulation with CCh (100 µM) or PMA+CCh (10 ng/ml and 100 µM, respectively) in cells transfected with siOrai1 or shTRPC1. Histogram shows % decrease in CCh-stimulated luciferase activities in shTRPC1- or siOrai1-treated cells (relative to that in mock-transfected control cells). Data were obtained in at least 3 separate experiments for each transcription factor.

To further establish whether the functional specificity of TRPC1 and Orai1 seen in short-term responses (i.e. nuclear translocation of NFAT) is also retained for long-term effects at the level of gene expression in the nucleus, we measured NFAT- or NFκB-driven luciferase activities. Such long-term effects would indicate that a [Ca^2+^]_i_ signal “memory” is retained even after the initial [Ca^2+^]_i_ elevation has declined. Both NFAT- and NFκB-driven luciferase (NFAT-luc and NFκB-luc, respectively) activities were clearly detected following 100 µM CCh treatment of cells (substantial variability was seen with lower [CCh], i.e. 10 and 1 µM, possibly due to the lower percentage of cells responding to CCh at these concentrations). Consistent with the findings shown in Figures 3 and 5, loss of Orai1 function, but not that of TRPC1, significantly abrogated CCh-stimulated increase in NFAT-dependent promoter activity (Figure 7). Thus, both early and late events in NFAT signaling are exclusively dependent on Orai1. In contrast, loss of either Orai1 or TRPC1 function significantly and severely reduced CCh-stimulated NFκB-driven promoter activity (Figure 7). We had earlier shown that Orai1-mediated Ca^2+^ entry is required for the assembly of functional TRPC1-STIM1 channels in the plasma membrane [27]. Therefore abrogating Orai1 function eliminates TRPC1 channel activity and accounts for the decrease in NFκB-luc in shTRPC1- or siOrai1-treated cells. Further, the similar levels of NFκB-luc activity measured in the two groups of cells suggest that TRPC1 is the primary determinant in the regulation of this transcription factor. In aggregate, our findings provide strong evidence for the functional specificity of Orai1 and TRPC1 channels in the regulation of Ca^2+^-dependent gene expression.

## Discussion

In some cell types, including salivary gland cells, more than one channel contributes to agonist stimulated [Ca^2+^]_i_ signals. It is not fully understood how cells decode [Ca^2+^]_i_ signals originating from multiple sources for the regulation of specific Ca^2+^-dependent functions. Variations in the pattern of individual [Ca^2+^]_i_ signals generated by the two channel types is most likely the primary determinant of the functional specificity of the channels in regulation of cell function. Here we have studied the contributions of endogenous TRPC1 and Orai1 to agonist-stimulated [Ca^2+^]_i_ signals in a single HSG cell. We show that Ca^2+^ entry via each channel generates a specific pattern of [Ca^2+^]_i_ elevation, with Orai1 controlling the generation of [Ca^2+^]_i_ oscillations and TRPC1 mediating sustained [Ca^2+^]_i_ elevation at higher [agonist] and contributing to the frequency of baseline [Ca^2+^]_i_ oscillations. Even more significant is the finding that the channels display functional specificity in the activation of Ca^2+^-dependent transcription factors and gene expression. Consistent with the oscillatory [Ca^2+^]_i_ signals generated by Orai1, NFAT translocation and NFAT-dependent gene expression were exclusively dependent on Orai1-mediated Ca^2+^ entry, without any contribution of TRPC1. Our data suggest that NFAT is strictly regulated by the [Ca^2+^]_i_ achieved locally near the Orai1 channel, likely due to localization of calmodulin-calcineurin-NFAT within the Orai1-associated microdomain, such that the Ca^2+^ entering via Orai1 can be locally sensed by the calcium sensor. Moreover, since Ca^2+^ entering into this microdomain via Orai1 rapidly rises to concentrations that exceed a threshold level required for activation, NFAT activation did not reflect global [Ca^2+^]_i_ changes achieved at the various stimulus intensities. We also show that NFAT activation follows an “all-or-none” mode of activation which is strictly dependent on Orai1; if an insufficient number of Orai1 channels is activated, NFAT dephosphorylation is not completed and nuclear translocation does not occur. However, we cannot rule out the possibility that at even higher level of stimulus (e.g. a more potent agonist or involving different receptor pathways) or if more channels were expressed, this pattern could vary and sustained [Ca^2+^]_i_ elevations could be induced by Orai1 (e.g. in lymphocytes or in HEK293 cells overexpressing Orai1+STIM1). In contrast to the regulation of NFAT, we show that NFκB is primarily regulated by TRPC1. Furthermore, we previously reported that Ca^2+^ entry via TRPC1, but not Orai1, is required for sustained activation of K_Ca_ in HSG cells as well as acinar cells isolated from mouse salivary glands [20]. However, it remains to be fully understood whether local [Ca^2+^]_i_ achieved near the TRPC1 channel or global [Ca^2+^]_i_ changes mediated by TRPC1 are involved in the activation of NFκB and K_Ca_ channel. In aggregate, these findings provide conclusive evidence that Orai1 and TRPC1 generate functionally specific local and global [Ca^2+^]_i_ signals.

An important determinant in the generation of local [Ca^2+^]_i_ signals is the clustering of the channels within the signaling microdomain [38], [39], [40], [41]. Furthermore, localization of other Ca^2+^ signaling components, such as Ca^2+^ pumps in the ER and plasma membrane or mitochondria, could also affect the amplitude as well as temporal and spatial aspects of [Ca^2+^]_i_ signals [5], [7], [31]. More recently, stimulation with different types of agonist has been shown to recruit different STIM proteins to activate Orai1 channel and generate different [Ca^2+^]_i_ signals that are decoded to induce NFAT-driven gene expression in RBL cells [32]. In this context, a recent report describes that distinct modes of Ca^2+^ signaling are triggered by Ca_v_1 and Ca_v_2 channels within the same neurons, which are differentially used for regulating gene expression [42]. Both Ca_v_1 and Ca_v_2 channels are activated following plasma membrane depolarization and employed the same calmodulin kinase (CaMK)-dependent pathway to activate CREB-dependent gene expression. Nonetheless, Ca^2+^ entry via both channels contributed to different pools of Ca^2+^ within a single neuron with Ca_v_1 contributing to the local [Ca^2+^]_i_ but Ca_v_2 to the global [Ca^2+^]_i_ increases. Interestingly, Ca_v_1 was generally clustered close to the puncta of βCaMKII, the predominant CaMKII isoform in neurons, while Ca_v_2 clusters were located supramicrons away. Another important point was that Ca^2+^ entry via Ca_v_2 channels was preferentially buffered by the ER and mitochondria. Hence, Ca_v_2-mediated Ca^2+^ signaling was dampened to a greater degree than Ca_v_1 and requires a greater depolarizing stimulus for channel activation [42]. The molecular components of the Ca^2+^ signaling microdomain associated with TRPC1 and Orai1 needs to be further investigated. It is important to note that while the present study has revealed the Orai1-dependent [Ca^2+^]_i_ changes that underlie CCh-stimulated [Ca^2+^]_i_ increases, we have not yet resolved the [Ca^2+^]_i_ signals generated by TRPC1 alone. We have identified the contributions of TRPC1 to the overall [Ca^2+^]_i_ increases, but since TRPC1 function obligatorily depends on Orai1, we currently do not have conditions where Orai1 function can be selectively inhibited following activation of both channels. Efforts are underway to sort out this rather complicated issue.

In conclusion, agonist stimulation of cells leads to activation of SOCE with contributions from both TRPC1 and Orai1 channels. While Ca^2+^ entry via TRPC1 modifies the amplitude and frequency of agonist-induced, Orai1-dependent, [Ca^2+^]_i_ signals, it does not have any impact on the local Ca^2+^ signals driven by Orai1. This was demonstrated by the exclusive role of Orai1 in regulation of NFAT, without any effect of global [Ca^2+^]_i_ increases or contributions due to TRPC1-mediated Ca^2+^ entry. Together our findings strongly demonstrate that distinct global and local [Ca^2+^]_i_ signals are generated by Orai1 and TRPC1 in a single cell, which are specifically decoded to activate different gene expression pathways. Moreover, stimulus intensity determines the number of each channel that is activated and consequently the magnitude of [Ca^2+^]_i_ achieved locally near the pore of either channel. Further studies will be required to measure the local [Ca^2+^]_i_ within each channel microdomain and identify the components involved in decoding the specific [Ca^2+^]_i_ signals generated by them, as well as the mechanisms utilized for acute and long-term regulation of cellular functions.

## Materials and Methods

### Cell Culture, Cell Transfection and Reagents

HSG and HEK293 cells were cultured as described previously [25], [27]. Sequences for the siSTIM1, siOrai1 (Thermo Fisher Scientific, Lafayette, CO) and shTRPC1 targeting to human STIM1, Orai1 and TRPC1 respectively were described previously [25], [27]. GFP-NFAT was obtained from Addgene (Cambridge, MA). Myc-STIM1-KK/EE [29] and Flag-Orai1E106Q [35], [36] were kind gifts from Drs. Shmuel Muallem (NIDCR, NIH, Bethesda, MD) and Anjana Rao (La Jolla Institute for Allergy and Immunology, La Jolla, CA) respectively. Lipofectamine RNAiMAX and Lipofectamine 2000 (Invitrogen, Grand Island, NY) were used for transfections of siRNAs and other DNA plasmids respectively. Cells were typically used 48 h post-transfection. The efficiency of protein knockdown (using siSTIM1, siOrai1 and shTRPC1), as well as the expression of STIM1-KK/EE and Orai1E106Q, in HSG and HEK293 cells have been reported previously [25], [26], [27]. All other reagents of molecular biology grade were obtained from Sigma Aldrich (St Louis, MO) unless mentioned otherwise.

### [Ca^2+^]_i_ Measurements

Fura-2 fluorescence was measured in single HSG cells as described previously [25], [27]. Cells were loaded with 2 µM Fura-2AM (Invitrogen) for 45 min at 37°C, fluorescence was recorded using a Polychrome V spectrofluorimeter (TILL Photonics, Victor, NY) and MetaFluor imaging software (Molecular Devices, Sunnyvale, CA). Each fluorescence trace (340/380 nm ratio) represents an average from at least 50 cells. For the bar graphs, data presented show change in Fura-2 ratio due to influx where the fluorescence value at 250 s or 350 s was subtracted from the baseline (F_t_−F_0_).

### Measurement of NFAT Translocation into the Nucleus

Translocation of NFAT in transfected HSG cells was observed using an Olympus IX81 motorized inverted microscope (Olympus, Center Valley, PA) and a TIRF-optimized Olympus Plan APO 60× (1.45 NA) oil immersion objective. Excitation was achieved [25] using the 488 nm laser for excitation of GFP, and emission detected using a Lambda 10-3 filter wheel (Sutter Instruments, Novato, CA) containing the 525-band pass (BP50m) filter. Images were collected using a Rolera EM-C^2^ camera (Q-Imaging, Surrey, BC) and the MetaMorph imaging software (Molecular Devices). MetaMorph was also used to measure the fluorescence intensity in the nucleus and cytoplasm before and after stimulation with CCh. Briefly, regions of interest were selected to obtain the values for their fluorescence intensities during a time course experiment. These values were then plotted using the Origin 8 software (OriginLab, Northampton, MA). Due to the low responsiveness of HSG cells to stimulation with 300 nM CCh, a 20× fluorescence objective was used to screen larger numbers of cells.

### Measurement of NFAT and NFκB Luciferase Activities

HSG cells were seeded at 15×10^3^ per well in a 96-well plate one day prior transfection. The shTRPC1 (0.25 µg/well) and siOrai1 (200 nM/well) constructs were transiently transfected into cells using Lipofectamine 2000 or RNAiMAX (Invitrogen), respectively, following manufacturer’s protocol. After 24 h, the firefly luciferase reporter constructs for NFAT (pGL4.30[*luc2P*/NFAT-RE/Hygro]) or NFκB (pGL4.32[*luc2P*/NF-κB-RE/Hygro]) were transfected with the renilla luciferase reporter construct (pGL4.74[*hRluc*/TK]; to monitor transfection efficiency) into HSG cells using Lipofectamine 2000 for another 24 h. All three luciferase constructs were obtained from Promega (Madison, WI). Cells were then left untreated or treated with CCh at various concentrations for 6 h at 37°C. For NFAT luciferase activity, cells were stimulated with CCh alone. For NFκB luciferase activity, cells were stimulated with CCh in the presence of PMA (10 ng/ml). Luciferase activities were determined using Dual-Glo Luciferase Assay, as per manufacturer’s instructions (Promega). Luminescence intensity was monitored in using the FLUOStar OMEGA microplate reader (BMG Labtech, Cary, NC). At least 3 separate experiments were performed using samples in triplicates. The firefly luciferase values were normalized to renilla luciferase values. All data were presented as fold-change relative to the vector control.

### Statistics

Data analysis was performed using Origin 7.0 (OriginLab). Statistical comparisons were made using Student’s t-test. Experimental values are expressed as mean±SEM. Differences in the mean values were considered to be significant at *p*<0.001.

## Supporting Information

Figure S1 **Effect of expressing TRPC1 and STIM1 on low [CCh]-induced Ca^2+^_i_ responses in HEK293 cells.** Baseline Ca^2+^_i_ oscillations in HEK293 cells following 1 µM CCh stimulation in control cells (**A**), cells expressing TRPC1+STIM1 (**B**) or STIM1-KK/EE (**C**), and control cells without Ca^2+^ present in the extracellular medium (**D**). Each trace is representative of ≥20 cells in at least 2 separate experiments.
(TIF)

Figure S2 **SOCE-driven [Ca^2+^]_i_ increases in HSG cells stimulated with high [CCh].** [Ca^2+^]_i_ responses induced by high [CCh] (100 µM) in control HSG cells (**A**) and cells expressing siSTIM1 (**B**), Orai1E106Q (**C**), siOrai1 (**D**), shTRPC1 (**E**), or STIM1-KK/EE (**F**). Each trace is representative of ≥50 cells in at least 3 separate experiments (**G**). Average data showing amplitude of [Ca^2+^]_i_ increase at t = 250 s (F_t_−F_0_). *** indicates a significant difference (P<0.001, n ≥ 80 cells).
(TIF)
