# Supplementary figures and images for: Distinct Contributions of Orai1 and TRPC1 to Agonist-Induced [Ca2+]i Signals Determine Specificity of Ca2+-Dependent Gene Expression
Source: PLoS One. 2012 Oct 24;7(10):e47146. doi: 10.1371/journal.pone.0047146 (PMC3480383; doi:10.1371/journal.pone.0047146)

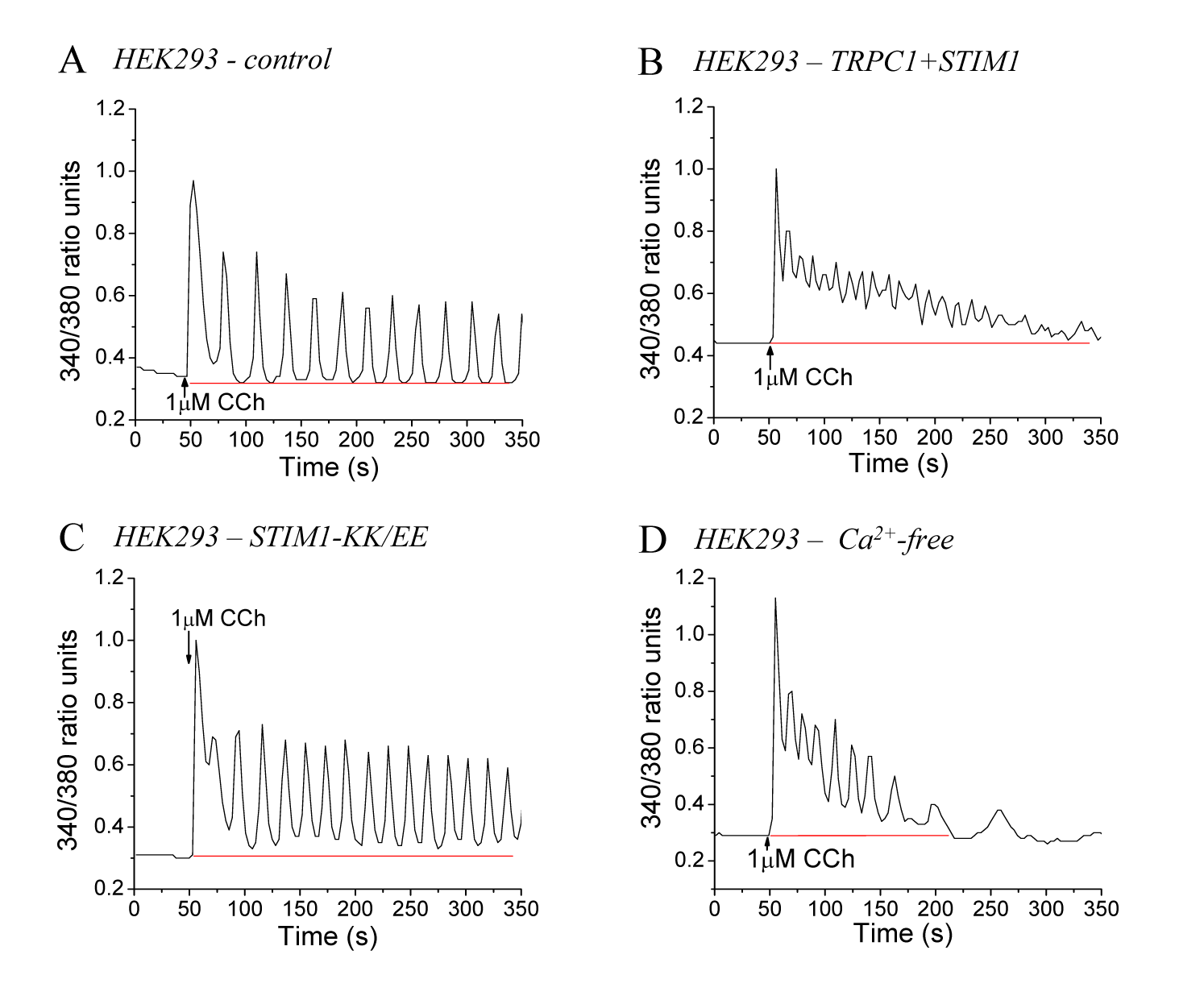

Supplement: Figure S1 — Effect of expressing TRPC1 and STIM1 on low [CCh]-induced Ca2+i responses in HEK293 cells. Baseline Ca2+ i oscillations in HEK293 cells following 1 µM CCh stimulation in control cells (A), cells expressing TRPC1+STIM1 (B) or STIM1-KK/EE (C), and control cells without Ca2+ present in the extracellular medium (D). Each trace is representative of ≥20 cells in at least 2 separate experiments. (TIF) [file pone.0047146.s001.tif]

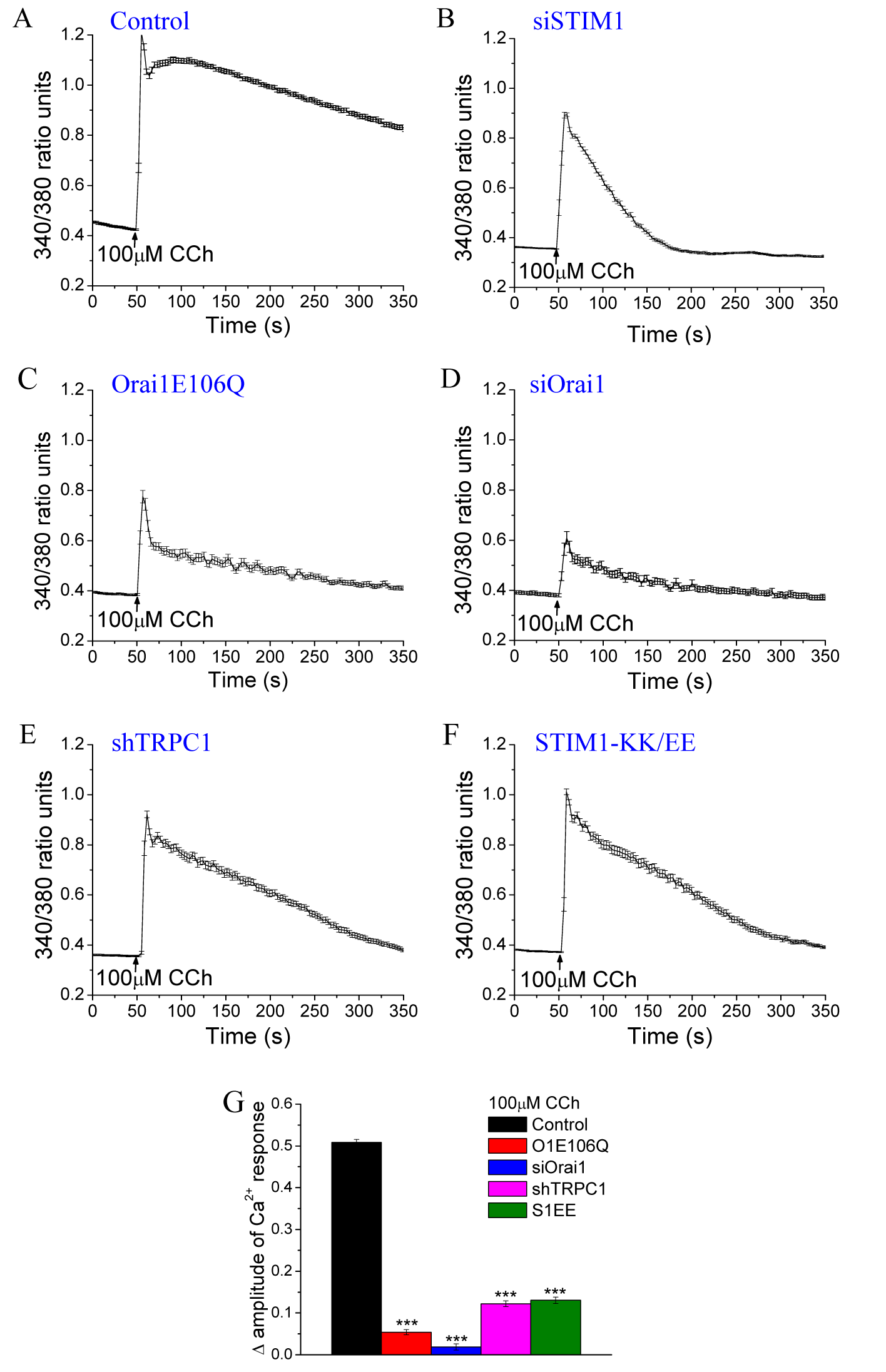

Supplement: Figure S2 — SOCE-driven [Ca2+]i increases in HSG cells stimulated with high [CCh]. [Ca2+]i responses induced by high [CCh] (100 µM) in control HSG cells (A) and cells expressing siSTIM1 (B), Orai1E106Q (C), siOrai1 (D), shTRPC1 (E), or STIM1-KK/EE (F). Each trace is representative of ≥50 cells in at least 3 separate experiments (G). Average data showing amplitude of [Ca2+]i increase at t = 250 s (Ft−F0). *** indicates a significant difference (P<0.001, n ≥ 80 cells). (TIF) [file pone.0047146.s002.tif]
